# Multiple breast tumors coexisting with chronic lymphocytic leukemia/small lymphocytic lymphoma: a rare case report

**DOI:** 10.1093/jscr/rjag423

**Published:** 2026-06-09

**Authors:** Ohoud Allinjawi, Salma Sait, Saleha Khan, Samratul Fuadah, Rana Ajabnoor, Rafat Abu Shakra, Nora Trabulsi

**Affiliations:** Department of Surgery, International Medical Center, 4238, Aruwais, 23214, Jeddah, Saudi Arabia; Department of Surgery, International Medical Center, 4238, Aruwais, 23214, Jeddah, Saudi Arabia; General Medicine Practice Program, Batterjee Medical College, Prince Abdullah Alfaisal St., 23819, Jeddah, Saudi Arabia; General Medicine Practice Program, Batterjee Medical College, Prince Abdullah Alfaisal St., 23819, Jeddah, Saudi Arabia; Department of Pathology, Faculty of Medicine, King Abdulaziz University, 3646 Almurtada, 22252, Jeddah, Saudi Arabia; Department of Pathology and Laboratory Medicine, International Medical Center, 4238, Aruwais, 23214, Jeddah, Saudi Arabia; Department of Surgery, Faculty of Medicine, King Abdulaziz University, 3646 Almurtada, 22252, Jeddah, Saudi Arabia

**Keywords:** breast cancer, chronic lymphocytic leukemia, small lymphocytic lymphoma, collision tumor, synchronous malignancy, case report

## Abstract

Concurrent presentation of multiple breast carcinomas with chronic lymphocytic leukemia/small lymphocytic lymphoma (CLL/SLL) is exceptionally rare in clinical practice. This report presents the case of a 77-year-old woman with two distinct right breast carcinomas. The patient underwent neoadjuvant carboplatin/paclitaxel chemotherapy with pembrolizumab, achieving partial radiologic response. Following neoadjuvant therapy, the patient underwent right breast mastectomy with sentinel lymph node biopsy, which was positive, prompting subsequent axillary lymph node dissection. Post-mastectomy pathology revealed residual ductal carcinoma with marked treatment effect, persistent lobular carcinoma, and multifocal CLL/SLL infiltration in breast tissue with metastasis to axillary lymph nodes. The final staging was ympT1c N1a and the patient recommended for adjuvant pembrolizumab plus capecitabine with radiotherapy for breast carcinoma and surveillance with positron emission tomography-computed tomography for CLL. The outcome underscores the importance of meticulous histopathological and immunohistochemical evaluation in distinguishing concurrent malignancies from metastatic or reactive lymphoid infiltrates.

## Introduction

Breast cancer is the most commonly diagnosed malignancy in women [1]. Most cases present as isolated tumors with well-established management and prognostic outcomes. In contrast, lymphoid neoplasms of the breast are rare, accounting for < 1% of all breast tumors, and may mimic or coexist with carcinoma, complicating diagnosis [1, 2].

Chronic lymphocytic leukemia/small lymphocytic lymphoma (CLL/SLL) involving the breast may present as a discrete mass or diffuse infiltration, often resembling primary breast cancer on imaging and clinical examination [3, 4]. This overlap increases the risk of misdiagnosis, particularly when carcinoma and lymphoma are present within the same breast or lymph node (LN) [5–7].

In rare instances, breast cancer and CLL/SLL occur synchronously as independent primary tumors, leukemic infiltration, or collision tumors, where both cell types coexist within a single lesion [5, 7]. These patterns carry distinct diagnostic and therapeutic implications, necessitating histopathology (HP) and immunohistochemistry (IHC) to differentiate epithelial from lymphoid disease [5, 8]. Population data suggest that patients with concurrent breast cancer and CLL have poorer outcomes, including reduced survival and higher rates of aggressive subtypes such as human epidermal growth factor receptor 2 (HER2)-positive disease and triple-negative breast cancer, possibly reflecting tumor biology or delayed diagnosis [9].

No standardized treatment guidelines exist for such synchronous presentations [6, 10]. Careful documentation of such cases is essential to guide management and long-term follow-up. Thus, we present the case of a 77-year-old woman with two distinct right breast carcinomas, triple-negative invasive ductal carcinoma (IDC) and hormone receptor-positive invasive lobular carcinoma (ILC), and concurrent multifocal infiltration of the breast and axillary lymph nodes by CLL/SLL.

## Case presentation

A 77-year-old woman presented with a right breast mass and a family history of breast cancer in a first-degree relative. Clinical staging was cT2N0. Imaging with mammography and ultrasound demonstrated two spiculated malignant lesions in the right breast: a larger lesion at the 7 o’clock position measuring 3.0 × 4.0 × 3.5 cm and a smaller lesion at 3 o’clock measuring 1.0 × 0.9 × 1.2 cm, without skin or nipple invasion.

**Figure 1:**
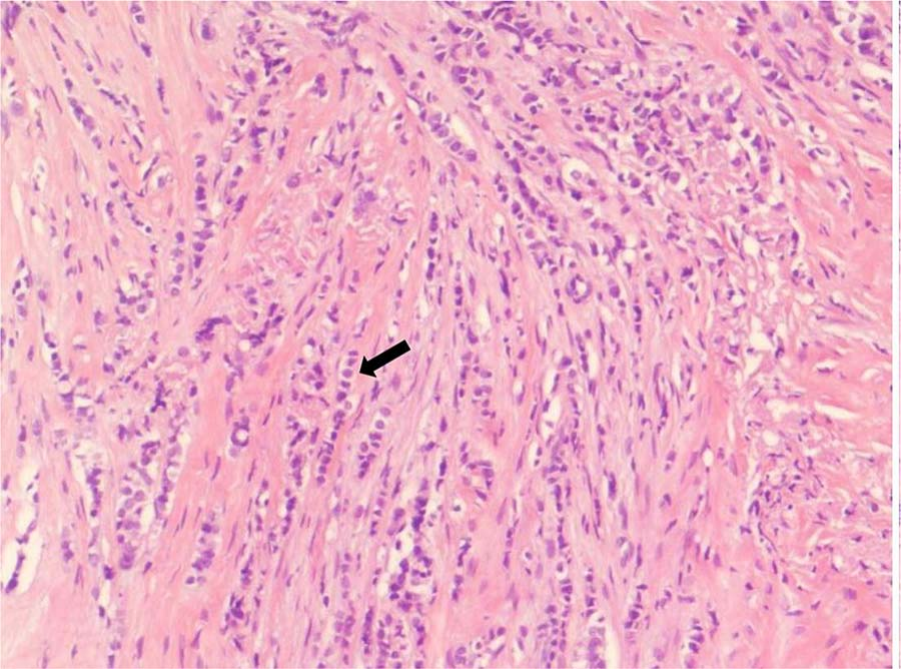
Core biopsy from the 3 o’clock right breast mass showing invasive lobular carcinoma. Tumor cells are arranged in the classic Indian files and show mild to moderate nuclear atypia (arrow) (HCE, 20×).

The right axilla showed prominent LNs with cortical thickening, while the left breast was normal. The radiologic impression was Breast Imaging Reporting and Data System BIRADS 5 (right) and BIRADS 1 (left). Ultrasound-guided core biopsies were obtained from both lesions and an axillary LN. Histopathology showed that the 7 o’clock lesion was a grade 3 triple-negative IDC, while the 3 o’clock lesion was an ILC that was estrogen receptor (ER)-positive, progesterone receptor (PR)-positive, and HER2-negative (Fig. 1). The axillary LN was negative for metastasis. Following a multidisciplinary team discussion, the patient received neoadjuvant chemotherapy with immunotherapy using carboplatin, paclitaxel, and pembrolizumab every 21 days.

**Figure 2:**
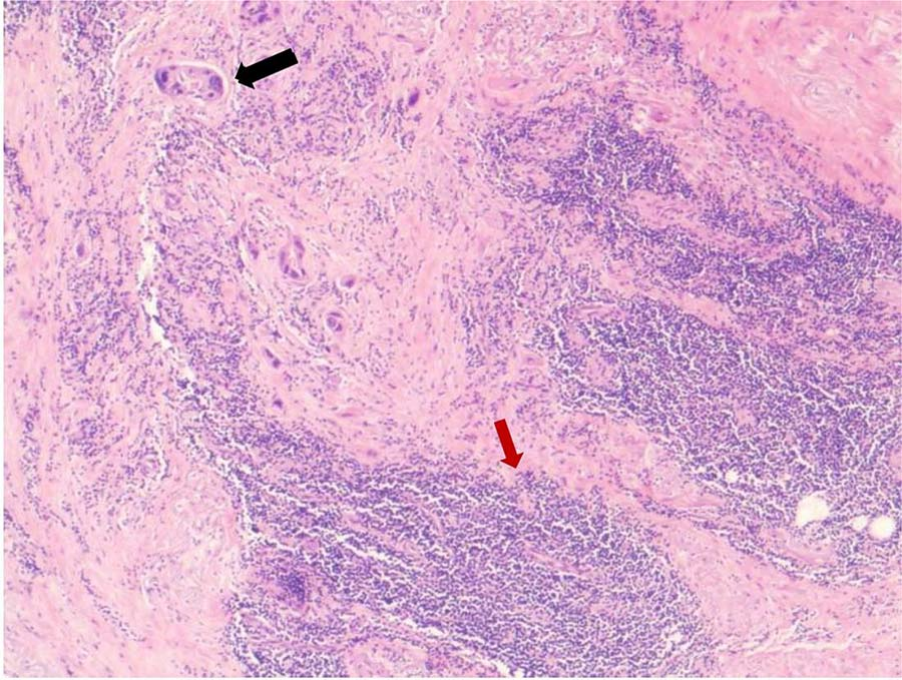
Mastectomy specimen post neoadjuvant treatment showing a few small residual foci of invasive ductal carcinoma (black arrow). Adjacent breast tissue shows dense small lymphocytic infiltrate consistent with small lymphocytic lymphoma (red arrow) (HCE,4×).

**Figure 3:**
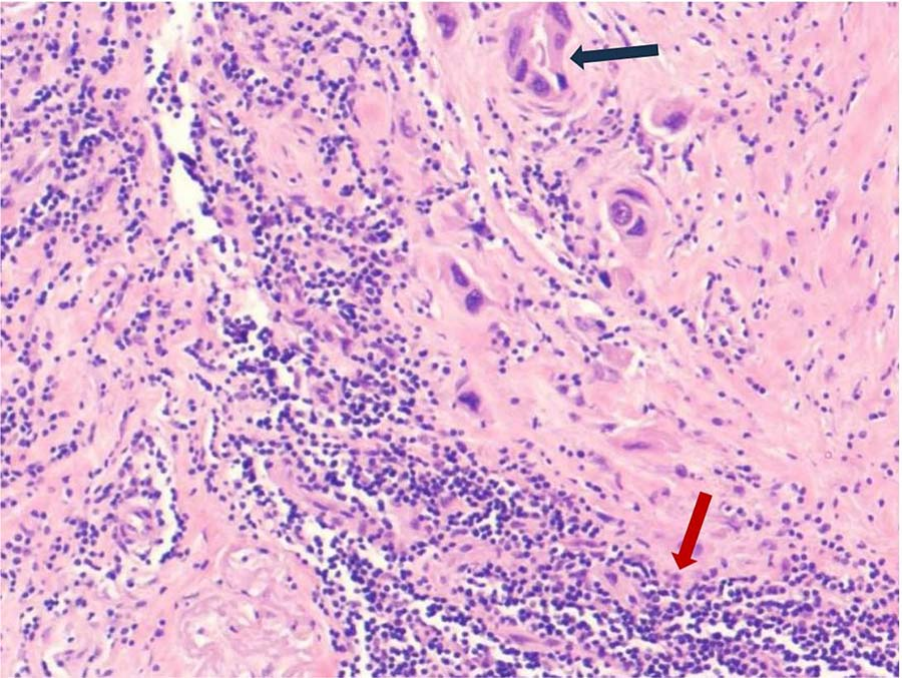
Mastectomy specimen post neoadjuvant treatment showing a few small residual foci of invasive ductal carcinoma (black arrow). Adjacent breast tissue shows dense small lymphocytic infiltrate consistent with small lymphocytic lymphoma (red arrow) (HCE,10×).

**Figure 4:**
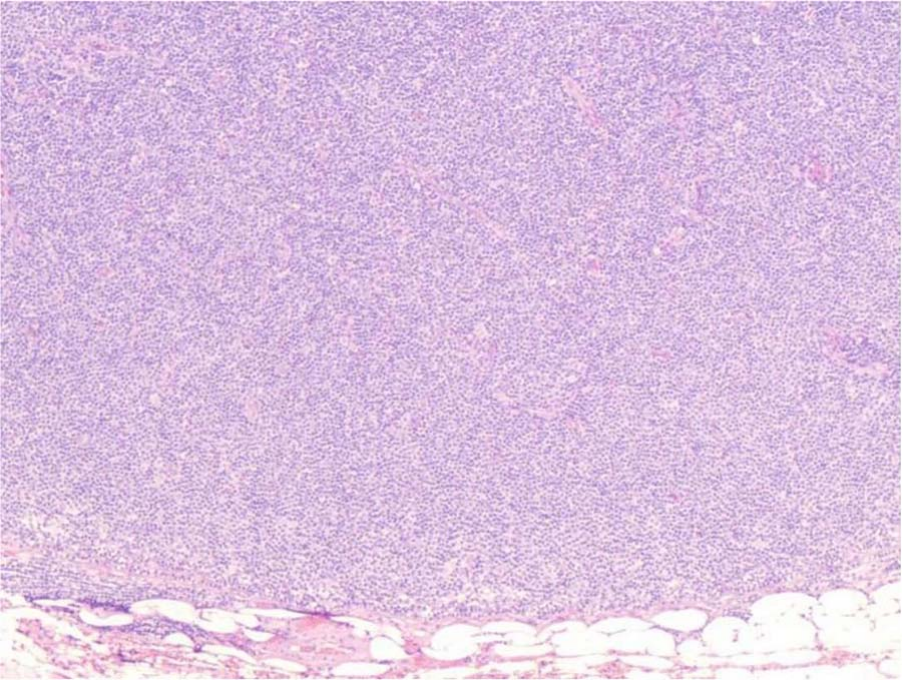
Axillary lymph node showing diffuse small lymphocytic infiltrate consistent with small lymphocytic lymphoma (HCE, 4×).

**Figure 5:**
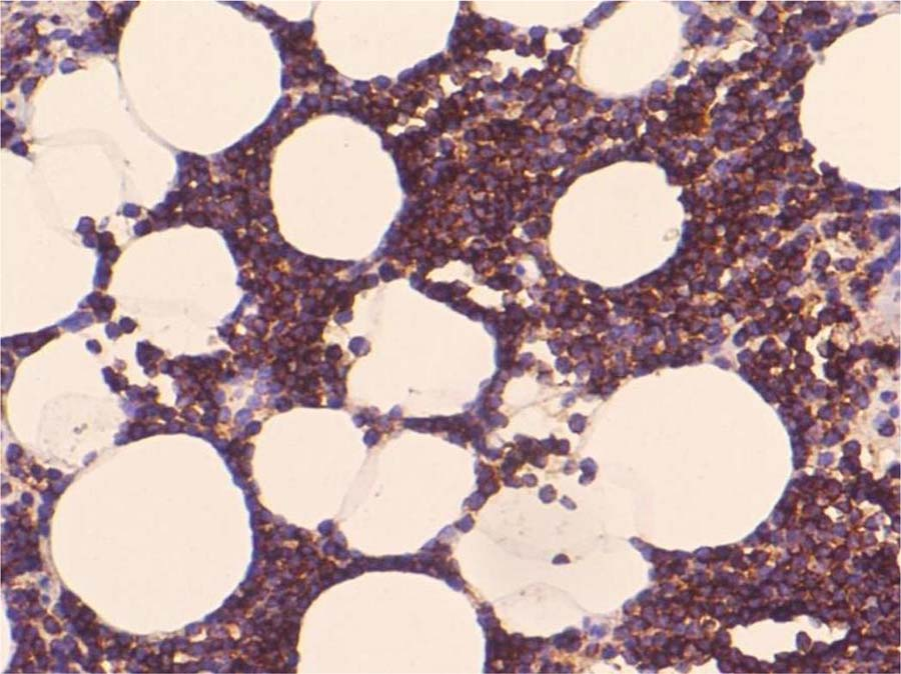
CD20 immunostain showing diffuse positive staining in the breast small lymphocytic infiltrate (20×).

**Figure 6:**
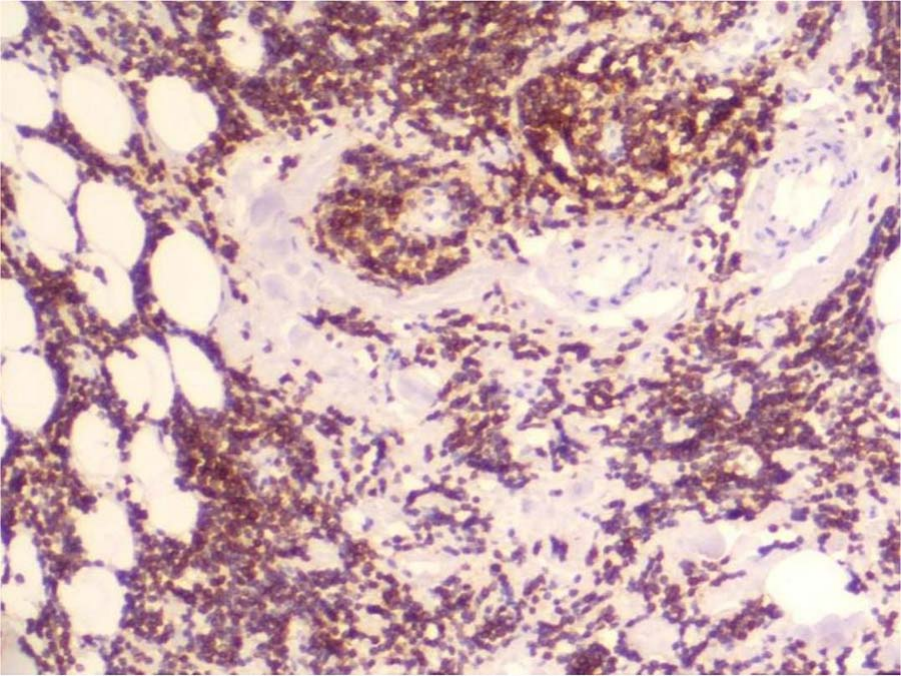
CD5 immunostain showing diffuse positive staining in the breast small lymphocytic infiltrate (10×).

**Figure 7:**
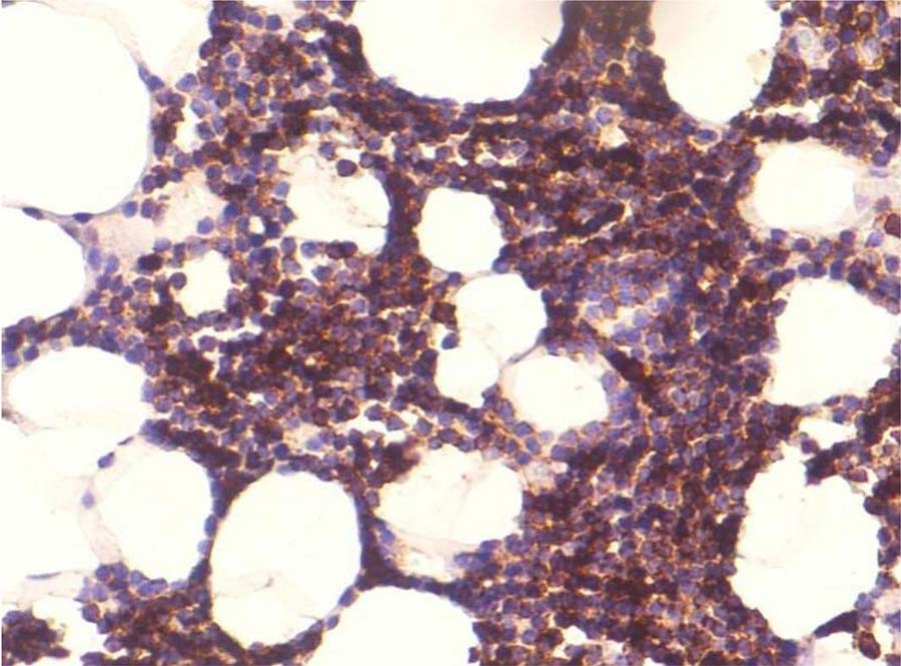
CD23 immunostain showing diffuse positive staining in the breast small lymphocytic infiltrate (20×).

After completion of neoadjuvant therapy, she underwent right mastectomy with sentinel lymph node biopsy. Intraoperative frozen section revealed metastatic involvement, prompting axillary lymph node dissection. Three sentinel LNs and 29 additional LNs were retrieved. Final HP demonstrated multiple coexisting pathologies. The IDC component showed a marked treatment response, with minimal residual tumor foci measuring 3.0 × 2.0 mm within fibrosis. In contrast, the ILC component showed minimal response and measured 1.7 × 1.4 × 1.2 cm, with ER and PR 100% positivity, HER2 negativity (score 0), and Ki-67 of 5%. Multifocal infiltration of breast tissue by CLL/SLL was also identified. LN analysis showed widespread CLL/SLL involvement, with only one LN positive for metastatic breast cancer (tumor deposit 4.0 × 3.0 mm) (Figs 2–4). Final staging was ympT1c N1a. IHC of the lymphoid component showed positivity for CD20, CD5, CD23, and BCL2, and negativity for CD3, CD10, BCL6, and cyclin D1, confirming CLL/SLL involvement (Figs 5–7).

Postoperatively, the patient received adjuvant pembrolizumab and capecitabine with radiotherapy, followed by endocrine therapy for ILC. The CLL/SLL was managed conservatively with surveillance due to its indolent nature. She tolerated treatment without complications. Follow-up included annual imaging with mammography, ultrasound, and positron emission tomography-computed tomography. Subsequent scans showed no metabolically active disease, with stable small LNs in cervical, left axillary, and mediastinal regions, and no evidence of recurrence in the operative bed.

## Discussion

Synchronous breast cancer and hematologic malignancy are rare but clinically significant. Patients with CLL/SLL have an increased risk of developing secondary solid tumors, including breast cancer, and often have poorer outcomes [11, 12]. Proposed mechanisms include immune dysregulation, prior treatments, shared genetic susceptibility, and advanced age. This case is notable for the coexistence of triple-negative IDC, hormone receptor-positive ILC, and multifocal CLL/SLL, representing a unique combination. Consistent with prior reports, the two carcinoma subtypes demonstrated differential chemosensitivity, with IDC showing marked regression and ILC showing limited response [13, 14].

Diagnosis remains challenging due to nonspecific imaging findings that cannot reliably distinguish breast cancer from lymphoid infiltration. Lesions typically appear as BIRADS 4 and 5 masses with variable characteristics; multiplicity or bilaterality may suggest lymphoma but lack specificity [2]. In this case, the lymphoid component mimicked reactive changes, highlighting the importance of a core biopsy and comprehensive IHC for accurate diagnosis and staging. Reported coexistence patterns include synchronous disease within the same specimen or LN, collision tumors, and CLL/SLL mimicking primary breast cancer [2, 4, 5]. This case reflects multiple patterns, emphasizing diagnostic complexity.

Management requires an individualized multidisciplinary approach. Current practice prioritizes treatment of the more aggressive malignancy, typically breast cancer, while monitoring indolent CLL/SLL [4, 5, 15]. Here, therapy targeted triple-negative IDC with surveillance of CLL/SLL. Although some regimens may have dual activity, data on combining targeted CLL therapies with breast cancer treatments are limited. Genetic and immunological factors may contribute, with reported associations including BRCA2 and NF1 mutations and immune-related polymorphisms [13, 15], though mechanisms remain unclear.

## Conclusion

This case highlights the complexity of synchronous breast cancer and CLL/SLL. It emphasizes the importance of accurate diagnosis using HP and IHC, recognition of differing tumor biology, and coordinated multidisciplinary management. Continued reporting of such cases is essential to improve understanding and guide future diagnostic and therapeutic strategies.
